# Draft genome sequences of 10 *Campylobacter jejuni* and *Campylobacter coli* isolates from human pediatric diarrhea patients admitted to a hospital and chicken sources in Bangladesh

**DOI:** 10.1128/mra.01354-25

**Published:** 2026-01-26

**Authors:** Mohimanul Islam, Bibi Amena, Tanvir Ahmad Nizami, Himel Barua, Sanjoy Kanti Biswas, Paritosh Kumar Biswas

**Affiliations:** 1 Department of Microbiology and Veterinary Public Health, Faculty of Veterinary Medicine, Chattogram Veterinary and Animal Sciences University 130057, Chattogram, Bangladesh; 2 Department of Microbiology, Chattogram Maa-O-Shishu Hospital Medical College, Chattogram, Bangladesh; University of Maryland Baltimore, Baltimore, Maryland, USA

**Keywords:** *Campylobacter coli*, *Campylobacter jejuni*, chicken, foodborne pathogen, genomics, pediatric diarrhea patients, zoonosis

## Abstract

*Campylobacter* zoonosis from poultry remains a global public health concern. We report on the genomic data of *Campylobacter jejuni* and *C. coli* isolated from hospitalized pediatric diarrhea patients and chicken ceca in Bangladesh to help provide deeper insight into genetic diversity and the possible association of pediatric campylobacteriosis with chicken-derived *Campylobacter*.

## ANNOUNCEMENT

*Campylobacter* is one of the leading bacterial causes of human gastroenteritis worldwide (1–3) primarily due to *Campylobacter jejuni* and *Campylobacter coli* (4). Poultry is a major reservoir and source of human infection (5), yet genomic data on these species from humans and poultry in Bangladesh remain scarce. Between July 2023 and May 2024, 32 *C*. *jejuni* and 39 *C*. *coli* were isolated from 120 chicken cecal samples collected from poultry farms and live bird markets in Chattogram, Bangladesh. Additionally, three *C*. *jejuni* and two *C*. *coli* were isolated from stool samples of 120 pediatric diarrhea patients admitted to Chattogram Ma-O-Shishu Hospital, Bangladesh (22.322970 N, 91.806230 E). Collected stool samples were selectively pre-enriched in Preston broth supplemented with 5% lysed horse blood (Oxoid, UK) using 1 mL of sample added to 9 mL of broth and incubated at 42°C for 24 h. Chicken cecal and pre-enriched stool cultures were streaked onto modified charcoal cefoperazone deoxycholate agar (mCCDA) (Oxoid, UK) and incubated at 42°C for 48 h. Suspected colonies were subcultured on 5% bovine blood agar (CM0271B; Oxoid, UK) for 48 h at 42°C, and species were identified by multiplex PCR (6). All incubation steps were performed under a microaerophilic atmosphere generated using CampyGen gas pack (Oxoid, UK) within an anaerobic jar (Oxoid, UK). A total of 10 isolates, five each of human and chicken origin, were randomly selected for whole-genome sequencing.

The selected isolates for the whole-genome sequencing were then re-cultured on 5% bovine blood agar (CM0271B; Oxoid, UK) for 48 h at 42°C under microaerophilic conditions. Genomic DNA was extracted from a single, well-isolated colony using the Monarch Spin gDNA Extraction Kit (New England Biolabs, USA), and DNA quality and quantity were assessed using a NanoDrop One spectrophotometer (Thermo Fisher Scientific, USA). DNA libraries were prepared from 1 ng of genomic DNA using the Nextera XT DNA Library Preparation Kit (Illumina, Inc., USA), and whole-genome sequencing was performed on an Illumina NextSeq 2000 platform (Illumina, Inc., USA) at the Poultry Research and Training Centre, Chattogram Veterinary and Animal Sciences University, Bangladesh, generating 2 × 150 bp paired-end reads. Quality assessment of raw reads was conducted using FastQC v0.12.1 (7), and low-quality bases and adapter sequences were trimmed with Fastp v0.23.4 (8). *De novo* genome assembly was performed using Shovill v1.1.0 (9) with SPAdes v3.14.1 (10), applying a minimum contig length threshold of 200 bp, and assembly quality was evaluated with QUAST v5.2.0 (11). Taxonomic confirmation was carried out using Kraken2 v2.1.3 (12), with all analyses performed within the Galaxy platform v24.1 (https://usegalaxy.org/) (13). Default parameters were used for all software, unless otherwise specified. Genome annotation was performed using the Prokaryotic Genome Annotation Pipeline (PGAP) v6.8 (14) at the National Center for Biotechnology Information (NCBI) following sequence submission. Detailed genomic characteristics are presented in Table 1. Sequence types (STs) and clonal complexes (CCs) were determined using the *C. jejuni/coli* PubMLST database (15). These genomic data provide valuable resources for comparative analyses of *Campylobacter* populations from poultry, human, and other sources.

**TABLE 1:** Metadata of *Campylobacter jejuni* and *C. coli* strains isolated from hospitalized pediatric diarrhea patients in Bangladesh and from ceca of chicken reared on farm or sold at live bird markets in the country

Strain ID	Species	Sample	Sampling date	SRA accession no.	GenBank accession no.	CC^*a*^	ST^*b*^	Genome size (bp)	GC content (%)	No. of contigs	N50 (bp)	No. of reads	Sequencing depth^*c*^ (×)	Genes (total), PGAP	No. of raw reads
MIH-1	*Campylobacter coli*	Human stool	28 April 2024	SRR30709100	JBHXWU000000000	828	828	1,906,907	31.02	107	141,439	1,854,312	231	2,092	2,007,703
MIH-5	*Campylobacter coli*	Human stool	05 May 2024	SRR30709096	JBHXWY000000000	828	830	1,779,228	31.11	25	169,998	1,699,615	214	1,906	1,914,739
MIH-2	*Campylobacter jejuni*	Human stool	12 May 2024	SRR30709099	JBHXWV000000000	353	9390	1,706,243	30.28	39	177,240	1,959,028	272	1,812	2,120,633
MIH-3	*Campylobacter jejuni*	Human stool	30 April 2024	SRR30709098	JBHXWW000000000	45	2109	1,646,108	30.42	22	202,422	1,709,250	246	1,726	1,837,828
MIH-4	*Campylobacter jejuni*	Human stool	23 May 2024	SRR30709097	JBHXWX000000000	42	4055	1,632,116	30.54	26	159,090	2,242,559	337	1,717	2,372,532
MIP-2	*Campylobacter coli*	Broiler chicken ceca	27 July 2023	SRR30709094	JBHXXA000000000	828	1770	1,816,515	31.06	79	233,348	1,794,358	244	1,950	1,894,021
MIP-3	*Campylobacter coli*	Layer chicken ceca	16 July 2023	SRR30709093	JBHXXB000000000	–^*d*^	12264	1,715,292	31.31	23	265,285	1,558,760	209	1,817	1,727,618
MIP-5	*Campylobacter coli*	Broiler chicken ceca	11 September 2023	SRR30709091	JBHXXD000000000	828	828	1,888,301	31	74	130,444	1,835,176	235	2,056	1,961,064
MIP-1	*Campylobacter jejuni*	Broiler chicken ceca	20 September 2023	SRR30709095	JBHXWZ000000000	353	9390	1,703,523	30.3	38	126,222	1,535,653	222	1,812	1,626,716
MIP-4	*Campylobacter jejuni*	Broiler chicken ceca	03 September 2023	SRR30709092	JBHXXC000000000	353	9390	1,718,369	30.26	55	158,717	1,649,755	232	1,832	1,768,317

^*a*^ CC, clonal complex.

^*b*^ ST, sequence type.

^*c*^ Reads were automatically subsampled to an effective depth of approximately 100× prior to assembly by Shovill (9).

^*d*^ The symbol “–” indicates that no clonal complex has been assigned for this sequence type according to the PubMLST database.

## Data Availability

All the 10 *Campylobacter* genome sequences are available under BioProject accession number PRJNA1162304, with individual accession numbers provided in Table 1.
